# Focal Serous Tubal Intra-Epithelial Carcinoma Lesions Are Associated With Global Changes in the Fallopian Tube Epithelia and Stroma

**DOI:** 10.3389/fonc.2022.853755

**Published:** 2022-03-21

**Authors:** Jingni Wu, Yael Raz, Maria Sol Recouvreux, Márcio Augusto Diniz, Jenny Lester, Beth Y. Karlan, Ann E. Walts, Arkadiusz Gertych, Sandra Orsulic

**Affiliations:** ^1^ Department of Obstetrics and Gynecology, David Geffen School of Medicine, University of California, Los Angeles, Los Angeles, CA, United States; ^2^ Biostatistics Research Center, Cedars-Sinai Medical Center, Los Angeles, CA, United States; ^3^ Jonsson Comprehensive Cancer Center, University of California, Los Angeles, Los Angeles, CA, United States; ^4^ Department of Pathology and Laboratory Medicine, Cedars-Sinai Medical Center, Los Angeles, CA, United States; ^5^ Department of Surgery, Cedars-Sinai Medical Center, Los Angeles, CA, United States; ^6^ Faculty of Biomedical Engineering, Silesian University of Technology, Zabrze, Poland; ^7^ Department of Veterans Affairs (VA) Greater Los Angeles Healthcare System, Los Angeles, CA, United States

**Keywords:** ovarian cancer, fallopian tube, secretory, ciliated, STIC, computational image analysis, computational pathology

## Abstract

**Objective:**

Serous tubal intra-epithelial carcinoma (STIC) lesions are thought to be precursors to high-grade serous ovarian cancer (HGSOC), but HGSOC is not always accompanied by STIC. Our study was designed to determine if there are global visual and subvisual microenvironmental differences between fallopian tubes with and without STIC lesions.

**Methods:**

Computational image analyses were used to identify potential morphometric and topologic differences in stromal and epithelial cells in samples from three age-matched groups of fallopian tubes. The Benign group comprised normal fallopian tubes from women with benign conditions while the STIC and NoSTIC groups consisted of fallopian tubes from women with HGSOC, with and without STIC lesions, respectively. For the morphometric feature extraction and analysis of the stromal architecture, the image tiles in the STIC group were further divided into the stroma away from the STIC (AwaySTIC) and the stroma near the STIC (NearSTIC). QuPath software was used to identify and quantitate secretory and ciliated epithelial cells. A secretory cell expansion (SCE) or a ciliated cell expansion (CCE) was defined as a monolayered contiguous run of >10 secretory or ciliated cells uninterrupted by the other cell type.

**Results:**

Image analyses of the tubal stroma revealed gradual architectural differences from the Benign to NoSTIC to AwaySTIC to NearSTIC groups. In the epithelial topology analysis, the relative number of SCE and the average number of cells within SCE were higher in the STIC group than in the Benign and NoSTIC groups. In addition, aging was associated with an increased relative number of SCE and a decreased relative number of CCE. ROC analysis determined that an average of 15 cells within SCE was the optimal cutoff value indicating the presence of a STIC lesion in the tubal epithelium.

**Conclusions:**

Our findings suggest that global stromal alterations and age-associated reorganization of tubal secretory and ciliated cells are associated with STIC lesions. Further studies will need to determine if these alterations precede STIC lesions and provide permissible conditions for the formation of STIC.

## Introduction

HGSOC accounts for ~70% of epithelial ovarian cancers, frequently presents at an advanced stage, and is associated with poor prognosis (1). Epidemiologic studies showed that the incidence of HGSOC increased with age and that advanced stage disease was more common in elderly women (1). Postmenopausal status and BRCA mutations were additional independent predictive factors for ovarian cancer (1, 2). However, the etiology of HGSOC is still unclear and discrete rate-limiting steps in ovarian cancer initiation that could inform early detection and prevention of ovarian cancer have not yet been identified.

A significant breakthrough in the understanding of HGSOC initiation was made upon close inspection of ovaries and fallopian tubes from risk reducing salpingo-oopherectomies (RRSO) in women at high risk for developing HGSOC due to inherited mutations in the BRCA genes (3–9). Early cancer lesions were detected in 5-10% of these women (3–9). The majority of the early lesions were present in the fallopian tube epithelia, rather than the ovary, indicating that the fallopian tube epithelial cells were the most likely precursors of HGSOC (10, 11).

The fallopian tube epithelium is composed of two major morphologic cell types: secretory and ciliated. In women of reproductive age, secretory and ciliated cells occur in an alternating pattern of 1-5 cells of the same type. Most early morphologic and genetic alterations have been observed in secretory cells but not in ciliated cells; hence, secretory cells are considered a precursor cell type for HGSOC (6–8, 12, 13). The lesion that morphologically most resembles early stage HGSOC is STIC (6–8, 12, 13). Cytohistologic characteristics of STIC lesions include: 1) nuclear changes, such as enlargement, hyperchromasia, atypia, and nucleolar prominence, 2) disorganization and loss of polarity, 3) epithelial tufting, 4) pleomorphism, 5) increased number of mitotic figures, and 6) the absence of ciliated cells (14–17). Additionally, STIC lesions usually exhibit the p53 signature and Ki67 >10% (18). Initially, only STIC lesions were thought to be the true fallopian tube precursor to HGSOC as they have similar morphologic and molecular features (19–21). However, ovarian cancer risk has been associated with other potential early serous precursors (ESPs) that have a benign appearance and a low proliferative index and do not satisfy the criteria of a STIC diagnosis (3, 9, 22–24). These ESPs include: serous tubal intraepithelial lesions (STIL) (17), which are characterized by lower Ki67 positivity and less cytologic atypia than STIC (16, 18), p53 signatures defined as a single layer of >12 consecutive secretory cells with aberrant p53 expression (7, 25, 26), benign secretory cell outgrowth (SCOUT) defined as a continuous run of >30 secretory cells (3, 5, 9, 22, 23, 27), and secretory cell expansion (SCE) defined as a continuous run of >10 secretory cells with normal p53 expression (3, 22, 23). It is unclear if these secretory cell alterations are precursors to STIC or independent precursors to HGSOC (24, 28).

If STIC is an intermediate step in the transition between ESP and HGSOC, we hypothesized that ESPs and other aberrations in epithelial and stromal cell architecture would be more common in the fallopian tubes of HGSOC patients with STIC compared to the tubes of HGSOC patients without STIC. Because age as well as the presence/absence of HGSOC are expected to influence the tubal epithelial and stromal architecture, our analyses were controlled for these variables.

## Materials and Methods

### Case Collection

After approval by the Cedars-Sinai Medical Center (CSMC) and University of California Los Angeles (UCLA) institutional review boards, H&E slides of fallopian tubes from a total of 89 cases were retrieved from the pathology files at CSMC and UCLA. The fallopian tubes had been surgically removed due to: 1) benign conditions (endometriosis, fibroids, or uterine prolapse), 2) RRSO (i.e. women with heritable BRCA mutations or first-degree family history of ovarian cancer), and 3) diagnosis of HGSOC. The presence or absence of STIC lesions was confirmed by a pathologist (AEW) using standard diagnostic criteria for STIC in H&E-stained sections with the aid of p53- and Ki67-immunoreactivity analyses in ambiguous cases (14–17).

### Digital Image Analysis

**Figure 1:**
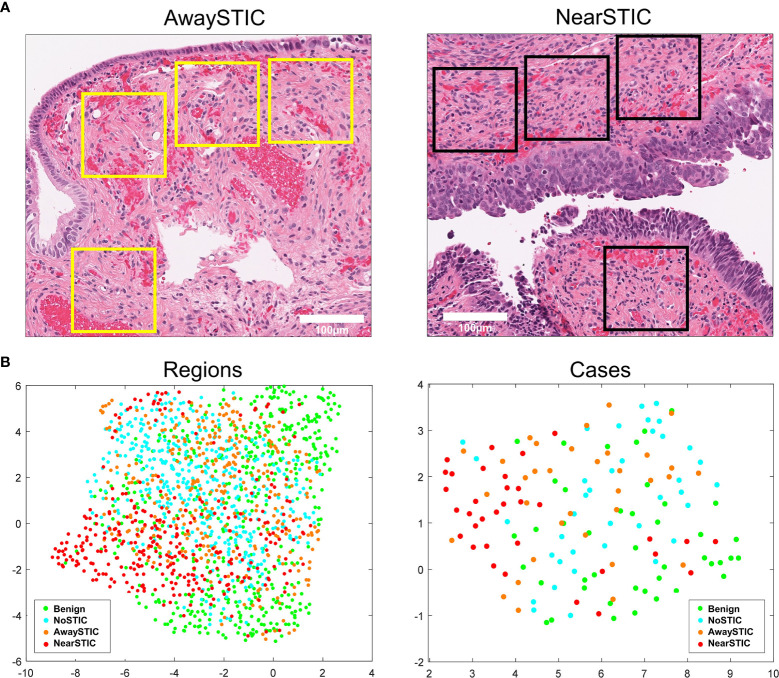
The fallopian tube stromal architecture changes both locally and globally in the presence of STIC lesions. **(A)** Yellow boxes illustrate stromal regions where tiles were extracted away from the STIC lesion (left panel) and black boxes illustrate stromal regions where tiles were extracted near the STIC lesion (right panel). **(B)** Low-dimensional UMAP representation of stromal morphometric features in regions and cases with benign fallopian tubes (Benign), uninvolved fallopian tubes from patients with HGSOC (NoSTIC), and fallopian tubes with STIC lesions and HGSOC. Image regions from fallopian tubes with STIC lesions were stratified based on proximity to the lesion (awaySTIC and nearSTIC). Expression in an individual case represents median expressions from regions in that case. The UMAP plots were generated using the run_umap.m function in MATLAB.

Glass slides were digitized at 20x magnification using the Aperio AT Turbo slide scanner (Leica Biosystems). The Aperio ImageScope v.12.4.3 digital slide viewer was used to extract stromal tiles (256 x 256 pixels). The tiles were positioned so that at least one side or corner of the tile was as close as possible to epithelial cells without the risk of including epithelial cells in the tile (**Figure 1A**). In the extracted tiles, stromal architecture was characterized using our previously developed panel of algorithms (29) which computes 45 segmentation-based fractal texture analysis features (SFTA) (30), 256 local phase quantization features (LPQ) (31), and 256 binarized statistical image features (BSIF) (32). When applied to H&E channels color-deconvoluted from a single H&E tile, the panel yielded 1114 features (557 eosin and 557 hematoxylin). Prior to feature extraction, the H&E tiles were color-normalized to reduce staining variability across cases (33). The tiles were divided into four groups: Benign, NoSTIC, AwaySTIC, and NearSTIC. NearSTIC was defined as a stromal tile subjacent to STIC (at least one side or corner of the image tile was adjacent to the epithelial cells in the STIC lesion) (**Figure 1A**). AwaySTIC was defined as a stromal tile subjacent to epithelial cells in a section of the tube that was >2 mm away from the STIC region demarcated by a pathologist. Within each case, the computed features were aggregated into vectors containing feature medians such that each case provided one vector per group. Feature vectors were z-scored and statistically analyzed using one-way ANOVA followed by the Tukey-Kramer test for multiple comparisons to identify differential morphologic features between the groups. The significance level α in the ANOVA was Bonferroni-corrected (α=0.05/1114). The topology of low dimensional representation of the feature vectors was visualized by the uniform manifold approximation and projection (UMAP) plot. The MATLAB implementation of UMAP was downloaded from https://www.mathworks.com/matlabcentral/fileexchange/71902-uniform-manifold-approximation-and-projection-umap.

**Figure 2:**
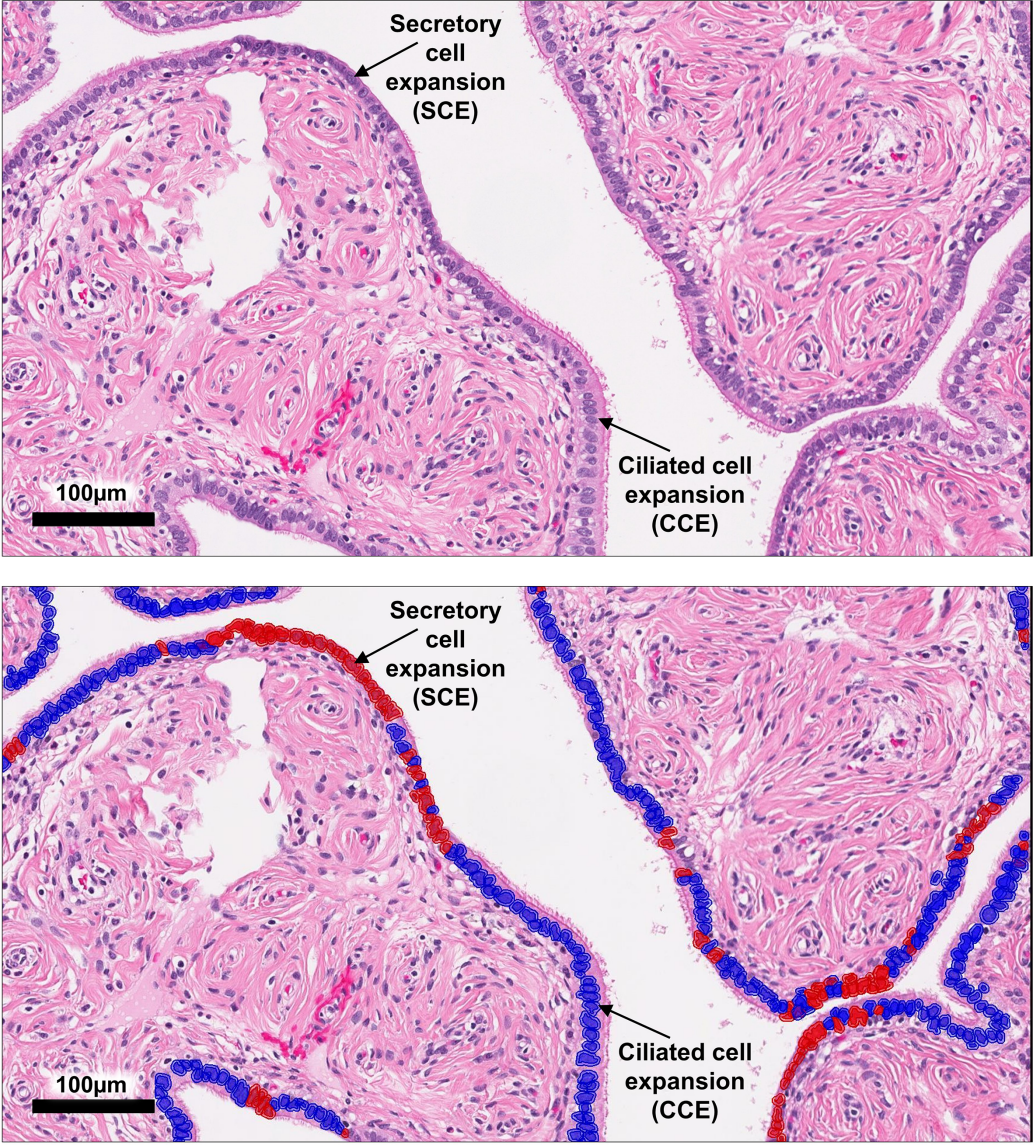
QuPath analysis of epithelial cells in the fallopian tube. A representative image of an ampulla region with SCE and CCE, which are defined as a monolayer of >10 contiguous secretory or ciliated cells uninterrupted by the other cell type. The top panel shows the original H&E image while the bottom panel shows epithelial cell classification after ROI selection, cell segmentation, and classifier application.

QuPath software (34) was used for H&E digital image analyses of the fallopian tube epithelium. Areas of the slide that contained a monolayer of visually recognizable secretory and epithelial cells were selected with a brush tool as regions of interest (ROIs). STIC lesions were excluded from the analysis. Cell segmentation algorithms, color deconvolution, and supervised classifiers were used as machine-learning methods. QuPath was used for cell identification and cell counting. We chose a random forest supervised classifier to recognize the cell types. The ciliated epithelial cells were identified by the presence of cilia on the apical surface while epithelial cells without cilia were considered to be secretory cells (**Figure 2**). After setting the appropriate cell segmentation and color deconvolution parameters in QuPath, we trained the object classifier to identify and count total epithelial cells, stromal cells, secretory cells, and ciliated cells. To account for differences in the number of epithelial cells available for examination on each slide, the relative number of SCE or CCE was defined as the number of monolayered contiguous runs of >10 secretory or ciliated cells divided by the total number of epithelial cells in all ROIs.

### Statistical Analysis of Epithelial Cell Topology

Continuous variables with a normal distribution were summarized using means and standard deviations (SD), otherwise they were summarized using medians and interquartile ranges. The categorical variables were summarized with rates using the Kruskal-Wallis test or the Fisher’s exact test. Two independent groups were analyzed by the Mann-Whitney test and two paired samples were analyzed by the Wilcoxon signed-rank test. Three or more independent groups were analyzed using the Kruskal-Wallis test. Variables with *P*< 0.05 by univariable analysis were included in the multivariable analysis using the log-transformed values. Multivariable linear regression was used to control for potential confounders, such as age, menopausal status, BRCA mutation status, and prior chemotherapy. The correlation between age and SCE, CCE, or cell number within the respective expansions was determined using the Spearman correlation method. The Receiver Operating Characteristic (ROC) curve was plotted to define the optimal cutoff values for the parameters of SCE and the cell number within SCE. All analyses were two-sided and the significance level was set at *P*< 0.05. The statistical analyses were conducted using SPSS 23.0 (SPSS Inc., Chicago, IL, USA).

### Sample Groups

The number of patients and the number of samples analyzed in each group depended upon the availability of the tube specimen that fulfilled specific criteria for individual analyses. In the majority of patients, only one tube and/or one tube region (fimbria or ampulla) fulfilled the criteria for a specific analysis. The following groups were analyzed:

- Stromal morphometric feature analyses: 1) Benign, 2) NoSTIC, 3) AwaySTIC, and 4) NearSTIC. No distinction was made between the fimbria and ampulla regions. Since the morphometric feature analysis was independent of clinical variables, each fallopian tube was considered a separate case even if it came from the same patient.
- SCE and CCE analyses: 1) Benign, 2) NoSTIC, and 3) STIC. The fimbria and ampulla regions were analyzed separately.
- Age-related analyses: 1) Benign, 2) NoSTIC, and 3) STIC. Only the fimbria region was analyzed.
- Confounder analyses: 1) Benign, 2) NoSTIC, and 3) STIC. The fimbria and ampulla regions were analyzed separately.
- ROC curve analyses: 1) STIC and 2) Benign+NoSTIC. The fimbria and ampulla regions were analyzed separately.

## Results

### Compared to Benign Fallopian Tubes, Local and Global Stromal Alterations Are Present in Fallopian Tubes With STIC Lesions and, to a Lesser Extent, Fallopian Tubes Without STIC Lesions From Patients With HGSOC

To study the stromal architecture associated with fallopian tubes, we extracted digital image tiles depicting fallopian tube stroma from women with benign gynecologic conditions (Benign group, n=43; 490 extracted tiles), fallopian tube stroma from women with HGSOC without STIC lesions (NoSTIC group, n=38; 446 extracted tiles), and fallopian tube stroma from women with HGSOC that contain STIC lesions (STIC group, n=34) (**Supplementary Figure 1A**). During extraction, the tiles in the STIC group were divided into two groups based on their proximity to the STIC lesions: AwaySTIC group (n=34; 338 extracted tiles) and NearSTIC group (n=34; 322 extracted tiles) (**Figure 1A** and **Supplementary Figure 1B**). There were no “visible” differences appreciated in the stroma upon review of the H&E stained slides or the stroma tile images from the four groups. However, ANOVA analysis revealed 250/1114 features with different means (*P*<4.48e-5) among the four groups of stromal tiles. Through the multiple comparison tests that followed ANOVA, we found features that distinguished Benign from NoSTIC (5 features), Benign from AwaySTIC (17 features), Benign from NearSTIC (208 features), NoSTIC from AwaySTIC (1 feature), NoSTIC from NearSTIC (188 features), and AwaySTIC from NearSTIC (54 features) (*P*<2.0e-4) (**Supplementary Figure 1C**). The presence of differentially expressed features is displayed through clustering of the region types and cases represented by UMAP feature embeddings (**Figure 1B**). Thus, although stromal changes in the fallopian tubes were not appreciable to the human eye with regular light microscopy, computational morphometric analysis revealed gradual global differences in the fallopian tube stroma from Benign to NoSTIC to AwaySTIC to NearSTIC (**Figure 1B**). These data suggest that STIC lesions are associated with local (NearSTIC) as well as global (AwaySTIC) stromal changes. The data also suggest that uninvolved fallopian tubes from HGSOC patients (NoSTIC) acquire global stromal changes that are in-between those in normal fallopian tubes from women with benign conditions (Benign) and those in fallopian tubes with STIC lesions (AwaySTIC).

### The Relative Number of SCE as Well as the Number of Cells Within SCE Are Higher in Fallopian Tubes With STIC Lesions

**Figure 3:**
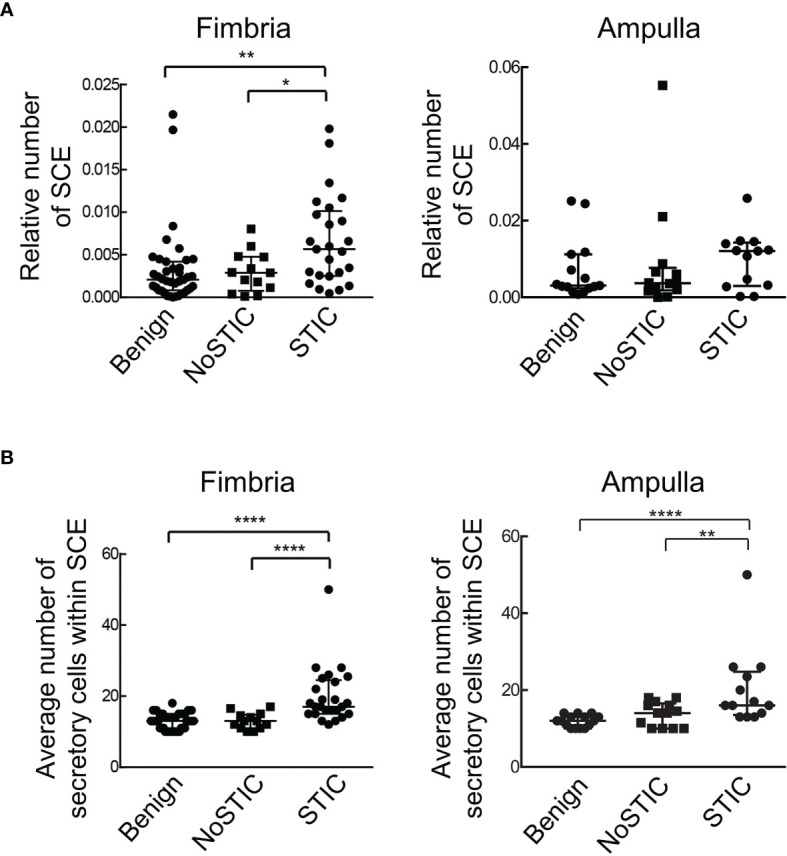
Fallopian tubes with STIC lesions exhibit a higher relative number of SCE as well as a higher number of secretory cells within SCE. **(A)** The relative number of SCE (number of SCE divided by the total number of epithelial cells in all ROIs on the slide). **(B)** The average number of secretory cells within SCE. Statistically significant differences were determined using the Kruskal-Wallis test; *P < 0.05; **P < 0.01; ****P < 0.0001. Benign: fallopian tubes from women with benign gynecologic conditions. NoSTIC: fallopian tubes without STIC lesions from women with HGSOC. STIC: fallopian tubes with STIC lesions from women with HGSOC.

The distribution of secretory and ciliated cells in samples with or without STIC lesions was evaluated using the QuPath digital image analysis software (34). Epithelial cell types were classified and quantitated in H&E slides in which monolayers of secretory and ciliated cells were recognizable by light microscopy. **Figure 2** shows a representative example of SCE and CCE (>10 contiguous cells of the same type). Areas of the fallopian tube that contained a STIC lesion were excluded from the analysis. The fimbria and ampulla regions were analyzed separately. For the analysis of the fimbria region, 77 cases (**Supplementary Table 1**) were divided into three groups: Benign (n=39), NoSTIC (n=13), and STIC (n=25). As shown in **Supplementary Table 1**, there was no significant age difference across the three groups. For the analysis of the ampulla region, 42 cases (**Supplementary Table 2**) were divided into three groups: Benign (n=16), NoSTIC (n=13), and STIC (n=13). As shown in **Supplementary Table 2**, there was no significant age difference across the three groups. In both the fimbria and ampulla regions, the relative number of SCE in the STIC group was higher compared to the Benign group and the NoSTIC group (**Figure 3A**) although statistical significance was only reached in the fimbria region. In both the fimbria and ampulla regions, the average number of cells within SCE was significantly higher in the STIC group compared to the Benign and NoSTIC groups (**Figure 3B**). Although the relative number of CCE and the average number of cells within CCE were higher in the STIC group compared to the Benign group (**Supplementary Figure 2**), these differences were not significant in subsequent multivariable analyses.

### Age Is Associated With an Increased Relative Number of SCE and a Decreased Relative Number of CCE in the Fallopian Tube Fimbriae

**Figure 4:**
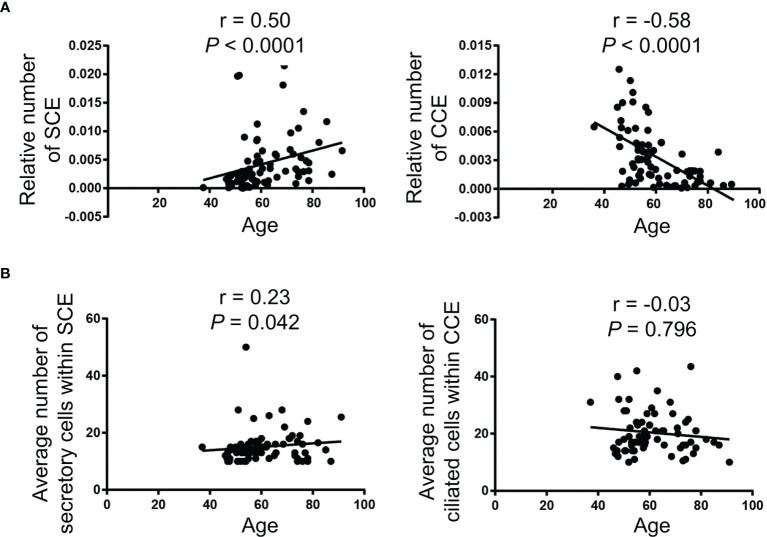
Age is correlated with an increased relative number of SCE and a decreased relative number of CCE in the fallopian tube fimbriae. **(A)** The relative number of SCE (left panel) and CCE (right panel) divided by the total number of epithelial cells in all ROIs on an individual slide. **(B)** The average number of secretory cells within SCE (left panel) and ciliated cells within CCE (right panel). The Spearman correlation (r) was used for the correlation analysis.

Spearman correlation analyses were performed to determine whether the numbers of SCE and CCE in the fimbriae were associated with age. With increasing age, the relative number of SCE was increased (r = 0.5, *P*<0.0001) while the relative number of CCE was decreased (r = -0.58, *P*<0.0001) (**Figure 4A**). There was a weak positive correlation between age and the number of cells within SCE, while the correlation between age and the number of cells within CCE was not significant (**Figure 4B**).

### Potential Confounders

Given the differences in menopausal status, BRCA mutation status, and prior chemotherapy among the Benign, NoSTIC, and STIC groups (**Supplementary Tables 1, 2**), multivariable linear regression models were developed to control for these potential confounders. The results of the analyses showed that fallopian tube fimbria from postmenopausal women had an increased relative number of SCE (*P*<0.0001, **Supplementary Figure 3A**) and a decreased relative number of CCE (*P*<0.05, **Supplementary Figure 3B**). The fallopian tube fimbria from postmenopausal women also had an increased average number of cells within SCE (*P*<0.01, **Supplementary Figure 3C**) but no significant difference in the average number of cells within CCE (**Supplementary Figure 3D**). Similarly, BRCA mutation status and prior chemotherapy treatment had an effect on the distribution of the tubal epithelial cells (**Supplementary Figure 3**). Even after controlling for these potential confounders, the relative number of SCE and the number of secretory cells within the SCEs were higher in the fallopian tube fimbriae with STIC lesions compared to the Benign and NoSTIC groups (**Supplementary Table 3**). Also, after considering confounders, we found differences in the secretory cell number within SCEs in the fallopian tube ampullae with STIC lesions (**Supplementary Table 4**). Other potential confounders, such as parity, duration of breast feeding, history of oral contraceptive use, and history of hormone therapy, were not evaluated in this study due to limited data availability. Of note, these factors were not significantly associated with STIC lesions in prior studies of large cohorts of patients (9, 35).

### Increased Number of Cells Within SCE Is a Potential Biomarker of STIC Lesions

**Figure 5:**
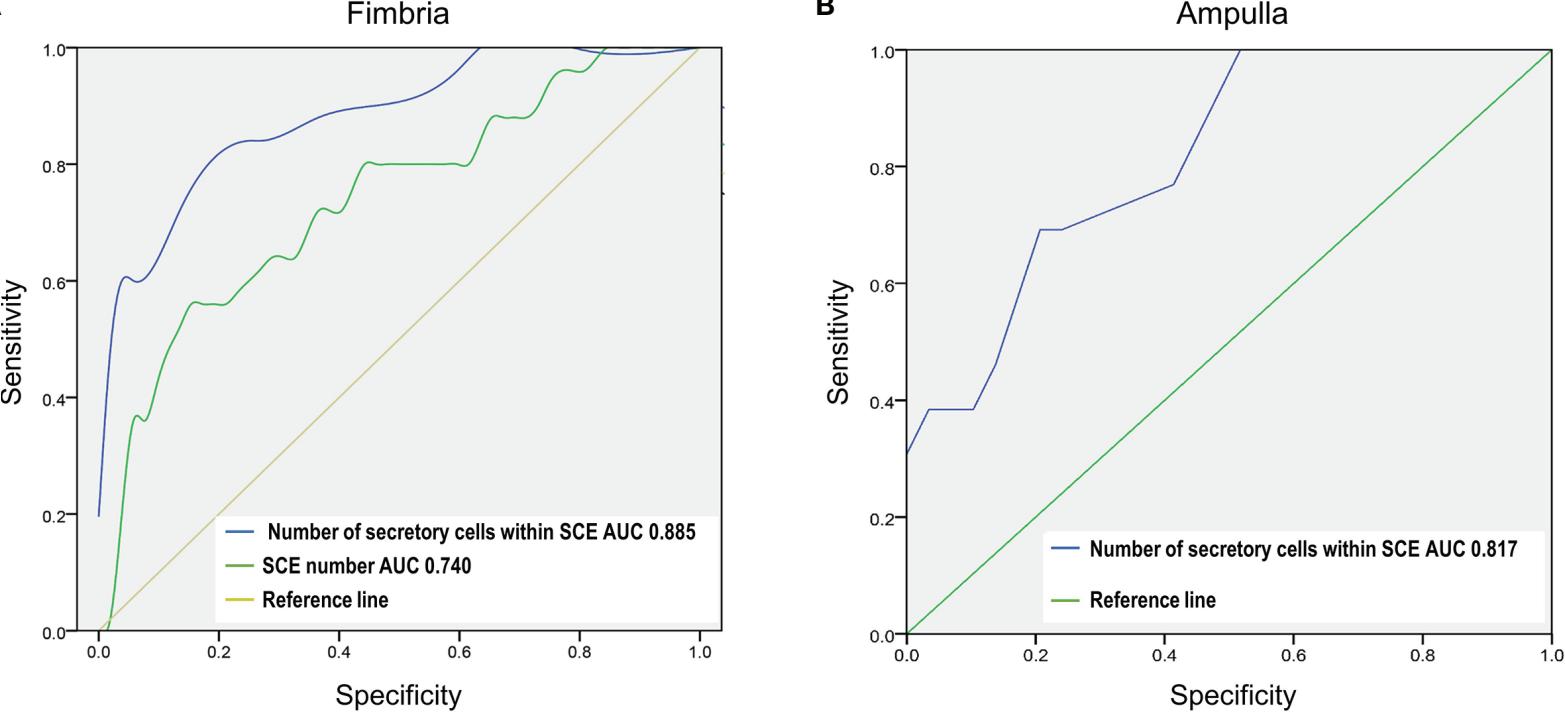
The number of secretory cells within a SCE is a potential biomarker of STIC lesions in the fallopian tube fimbria and ampulla. The ROC test was used to assess the diagnostic potential of global fallopian tube histopathology in identifying fallopian tubes with STIC lesions (STIC vs Benign+NoSTIC). **(A)** ROC curve analysis of the relative number of SCE and the number of secretory cells within SCE in the fimbria region. **(B)** ROC curve analysis of the number of secretory cells within SCE in the ampulla region.

Despite the development and validation of algorithms for STIC (14–17), the identification of STIC lesions remains challenging due to subjectivity and technical limitations. It has been shown that a review of slides by multiple pathologists increased the diagnosis of STICs by 50% while additional tissue sampling more than doubled the number of identified STICs (9). We conducted ROC curve analyses to evaluate whether the relative number of SCE or the number of cells within SCE could be used as biomarkers for easier identification of STIC lesions (STIC group compared to Benign+NoSTIC groups). The analyses showed that the number of cells within SCE can identify fallopian tube fimbriae with STIC lesions with an area under the ROC curve (AUC) of 0.885 (cutoff value=15, sensitivity=84%, specificity=75%;* P*< 0.0001; 95% CI: 0.806-0.965) in the fimbria region (**Figure 5A**) and 0.817 (cutoff value=15, sensitivity=69%, specificity=78%;* P*< 0.001; 95% CI: 0.619-0.861) in the ampulla region (**Figure 5B**). Additionally, we showed that in the fimbria region, the relative number of SCE can discriminate between the fallopian tubes with and without STIC lesions with an AUC of 0.740 (cutoff value=0.005, sensitivity=56%, specificity=86.5%;* P*< 0.001; 95% CI: 0.686-0.947) (**Figure 5A**).

## Discussion

HGSOC is the most lethal gynecologic cancer, largely due to delayed symptom presentation and the lack of effective early detection strategies. A poor understanding of the initiating events in ovarian cancer has significantly hampered efforts toward early detection and prevention. In the past two decades, detection of various morphologic and molecular changes in fallopian tube secretory cells, classified as STICs, STILs, p53 signatures, SCOUTs, SCEs and other ESPs, led to the hypothesis that HGSOC originates in the fallopian tube (3–11). Until recently, only STIC lesions were considered to be bona fide early precursors to HGSOC because of the existence of matching STIC and HGSOC site-specific TP53 mutations which strongly suggested the same lineage identity (20). However, the recent discovery of matching site-specific TP53 mutations between other ESPs and HGSOC as well as associations between ESPs and the increased risk of HGSOC (3, 22, 23) led to the “precursor escape” theory in which ESPs undergo malignant transformation after shedding into the peritoneal cavity, thus bypassing the formation of STIC lesions in the fallopian tube as a required intermediate step to HGSOC (24). Genomic profiling did not reveal significant differences between HGSOC with and without STIC lesions (10).

We addressed a different question: Is the presence of a STIC lesion associated with global changes in the tubal stroma and epithelium? Our computational morphometric feature analyses of the fallopian tube stroma from HGSOC patients revealed distinguishing morphometric features between fallopian tubes with and without STIC lesions. We also found that fallopian tubes with STIC lesions had an increased relative number of SCE as well as an increased number of cells within SCE. Together, these data suggest that STIC lesions are associated with global changes in the fallopian tube. Although it is unclear if these changes precede, occur concomitantly with, or are a consequence of STIC lesions, it is more likely that global alterations in the fallopian tube provide a favorable microenvironment for STIC formation than focal STIC lesions inducing global changes in the tube. Future studies are needed to determine whether these STIC-associated global topologic alterations also exhibit molecular changes that initiate STIC formation and could serve as targets for prevention. Of note, although the morphology of fallopian tube epithelia from HGSOC patients without STIC lesions resembled that of benign fallopian tubes, our data suggest that subtle architectural changes in the stroma precede HGSOC in the peritoneal cavity or that HGSOC can exert a long-distance field effect on the otherwise uninvolved fallopian tube stroma.

After examining the distribution of secretory and ciliated cells in fallopian tubes with or without STIC lesions, we further explored the relationship between the presence of expansions of secretory and ciliated cells and age. Epidemiologic studies have shown that the risk of HGSOC increases with age and that HGSOC is predominantly a disease of postmenopausal women (31, 32). Consistent with previously published studies (3, 36), we found that the number of cells within SCE increases with age while the number of cells within CCE decreases with age. The increase in the prevalence of p53 signatures with age in fallopian tubes with STIC lesions has been previously demonstrated (37, 38). However, to our knowledge, there are no publications that have determined the distribution of SCE and CCE alterations associated with age in the presence or absence of a STIC lesion. Most of the specimens in our study were obtained from postmenopausal women in order to minimize the influence of the follicular phase, which has been shown to markedly influence the proliferation and morphology of fallopian tube epithelial cells (39). Importantly, we showed that after controlling for age and menopausal status, the relative number of SCE as well as the number of cells within SCE were higher in the fallopian tube fimbria with STIC lesions.

Computational image analysis has advantages as well as disadvantages. Two major advantages are the ability to identify features not appreciable by conventional light microscopy and the ability to conduct rapid quantitative analyses. A disadvantage, however, is that machine learning-assisted cell segmentation and phenotype determination may not be as precise as pathologic examination and depends on careful manual selection of ROIs comprising monolayer runs of epithelial cells devoid of out-of-focus areas and artefacts. Nevertheless, we obtained concordant results in cases where we manually counted the numbers of secretory and ciliated cells within SCE and CCE, respectively.

While most prior histopathologic characterizations of fallopian tubes from patients with HGSOC focused on precursor lesions and essentially ignored other portions of the fallopian tube epithelia, we explored global histopathologic changes in the epithelial and stromal compartments in fallopian tubes with and without STIC lesions. This holistic view of fallopian tubes could and assist in identifying STIC lesions and assessing cancer risk. Currently, precursor lesions in fallopian tubes removed from women with a genetic predisposition to ovarian cancer are identified by trained pathologists. Since visual morphologic changes in precursor lesions are subtle, interpretations by pathologists are unavoidably subjective (9, 40). Yet, it is important to establish the correct diagnosis because women with isolated premalignant lesions in the fallopian tube are at increased risk of developing carcinomatosis, presumably because cancer cells have spread from the precursor lesion to other organs in the peritoneal cavity prior to preventive surgery. We anticipate that the global alterations in the tubal epithelia and stroma that we report here will assist pathologists in correctly classifying ambiguous cases. Additionally, a careful consideration of the global changes in the fallopian tube with STIC lesions could simplify the current protocol for pathologic examination of fallopian tubes. Since early cancer lesions are typically microscopic, entire fallopian tubes are cut into thin sections using the SEE-FIM (sectioning and extensive examination of the fimbria) dissection protocol with the distal fimbriae cut sagittally at 1 mm intervals (21). This can yield a large number of slides that need to be reviewed by a pathologist to potentially identify one section with an early cancer lesion. Even this meticulous method of sectioning might miss early cancer lesions if they occur within the 1 mm region between sections (9). Since our results show that fallopian tubes containing a STIC lesion exhibit a greater number of cells within SCE even after controlling for potential confounders, such as menopausal status, chemotherapy, and age, we suggest that SCEs containing more than 15 secretory cells should prompt a review of available sections and further microscopic examination of deeper levels of the fallopian tube paraffin blocks for features of a microscopic STIC lesion. Future validation and clinical standardization of cutoff parameters could improve risk stratification and personalization of preventive strategies for women with a predisposition to HGSOC.

To our knowledge, this is the first study of morphometric changes in the fallopian tube stroma associated with a STIC lesion. To date, only two studies have addressed the stromal changes associated with precursor lesions in the fallopian tube. One study measured changes in sulfated chondroitin sulfate expression in the extracellular matrix (41) while the other study measured the degree of collagen alterations by second harmonic generation microscopy (42). Both techniques revealed computationally discernable gradual differences in the fallopian tube stroma proceeding from normal tissue to STIC lesions to HGSOC.

Our study does not suggest that STIC is a requisite intermediate step to HGSOC, as about half of HGSOC and primary peritoneal cancers develop without any evidence of STIC lesions in the fallopian tubes. Rather, we have shown that in patients with HGSOC, the presence of a STIC is associated with other global alterations in the fallopian tube. It is possible that such alterations provide a permissible microenvironment for the development of STIC lesions, however, the underlying molecular mechanism remains unknown. A higher proliferation rate in the fallopian tube mucosa could explain the increased numbers of SCE and cells within SCE in the presence of a STIC lesion. Another factor that could contribute to an increased chance of STIC formation is a defect in an apoptotic mechanism to purge transformed cells. Future investigations and the elucidation of cellular and molecular processes that promote and inhibit the formation of premalignant lesions in the fallopian tube will facilitate the development of markers for early detection as well as the identification of rate-limiting events in the early stages of cancer development.

## Data Availability Statement

The original contributions presented in the study are included in the article/**Supplementary Material**. Further inquiries can be directed to the corresponding author.

## Ethics Statement

The studies involving human participants were reviewed and approved by Cedars-Sinai Medical Center Institutional Review Board and The UCLA Office of the Human Research Protection Program. Written informed consent for participation was not required for this study in accordance with national legislation and institutional requirements.

## Author Contributions

JW designed the project, conducted computational image analyses of the fallopian tube epithelial cells, performed statistical analyses of the data, and wrote the manuscript. YR and MR contributed to the image data analysis. MD conducted statistical analyses of the image data of the fallopian tube stroma. JL and BK contributed samples and annotated clinical data. AW reviewed the histologic slides, confirmed clinical diagnoses, and outlined STIC lesions on the slides. AG conducted computational image analyses of the fallopian tube stroma. SO conceived the project and contributed to the writing of the manuscript. All authors contributed to manuscript revisions. All authors contributed to the article and approved the submitted version.

## Funding

This research was supported by the Veterans Administration Merit Award VA-ORD BX004974 to SO; China Scholarship Council (No. 201806370108) to JW; to the Tina’s Wish Rising Star Award to MR; the Foundation for Women’s Cancer (FWC) Laura Crandall Brown Foundation Ovarian Cancer Early Detection Research Grant, the Rivkin Center Grant, and the Iris Cantor-UCLA Women’s Health Center/UCLA National Center of Excellence in Women’s Health Pilot Research Project NCATS UCLA CTSI grant UL1TR001881 to YR; and the Initiative of Excellence - Research University - a grant program at Silesian University of Technology (year 2021, no.07/010/SDU/10-21-01) to AG.

## Conflict of Interest

The authors declare that the research was conducted in the absence of any commercial or financial relationships that could be construed as a potential conflict of interest.

## Publisher’s Note

All claims expressed in this article are solely those of the authors and do not necessarily represent those of their affiliated organizations, or those of the publisher, the editors and the reviewers. Any product that may be evaluated in this article, or claim that may be made by its manufacturer, is not guaranteed or endorsed by the publisher.
