# Supplementary material for: Focal Serous Tubal Intra-Epithelial Carcinoma Lesions Are Associated With Global Changes in the Fallopian Tube Epithelia and Stroma
Source: Front Oncol. 2022 Mar 21;12:853755. doi: 10.3389/fonc.2022.853755 (PMC8977528; doi:10.3389/fonc.2022.853755)
Supplement: Supplementary file 1 [file DataSheet_1.pdf]

Supplementary Table 1. Characteristics of patients whose fimbria region of the fallopian tube was examined.

Characteristic	Benign n=39	NoSTIC n=13	STIC n=25	*P* value		
				NoSTIC *vs.* Benign	STIC *vs.* Benign	STIC *vs.* NoSTIC
**Age in years**						
Median (range)	56 (46-87)	65 (37-82)	58 (46-91）	NS [a]	NS [a]	NS [a]
**Menopause**						
Premenopausal	9 (23.1%)	2 (15.4%)	0 (0%)	NS [b]	0.009 [b]	NS [b]
Postmenopausal	30 (76.9%)	11 (84.6%)	25 (100%)			
**Chemotherapy**						
Unknown	3 (7.7%)	6 (46.2%)	3 (12%)	<0.0001 [b]	0.021 [b]	0.045 [b]
No	34 (87.2%)	3 (23.1%)	15 (60%)			
Yes	2 (5.1%)	4 (30.7%)	7 (28%)			
**BRCA Mutation**						
Not tested	5 (12.8%)	7 (53.8%)	5 (20%)	0.002 [b]	NS [b]	0.046 [b]
Negative	8 (20.5%)	4 (30.8%)	7 (28%)			
Positive	26 (66.7%)	2 (15.4%)	13 (52%)			

Statistical tests were performed with a Kruskal-Wallis test[a] or a Fisher's Exact Test[b]. Values are considered significant if $P<0.05$. Age is expressed as a median (interquartile range). Menopause, chemotherapy, and BRCA mutations were expressed as percentiles.

Benign: Normal fallopian tubes from prophylactic surgeries or benign conditions from women without STIC or cancer.

NoSTIC: Fallopian tubes from women who have ovarian cancer but the examined fallopian tubes do not contain transformed cells.

STIC: Fallopian tubes with STIC lesions from women who have ovarian cancer. NS, not significant.

Supplementary Table 2. Characteristics of patients whose ampulla region of the fallopian tube was examined.

Characteristic	Benign n=15	NoSTIC n=13	STIC n=13	*P* value		
				NoSTIC *vs.* Benign	STIC *vs.* Benign	STIC *vs.* NoSTIC
**Age in years**						
Median (range)	63 (48-87)	64 (37-82)	64 (51-75）	NS [a]	NS [a]	NS [a]
**Menopause**						
Premenopausal	0 (0%)	3 (23.1%)	0 (0%)	NS [b]	NS [b]	NS [b]
Postmenopausal	15 (100%)	10 (76.9%)	13 (100%)			
**Chemotherapy**						
Unknown	3 (20%)	8 (61.5%)	3 (23.1%)			
No	12 (80%)	2 (15.4%)	6 (46.2%)	0.001 [b]	NS [b]	NS [b]
Yes	0 (0%)	3 (23.1%)	4 (30.7%)			
**BRCA Mutation**						
Not tested	4 (26.7%)	8 (61.5%)	4 (30.8%)			
Negative	4 (26.7%)	3 (23.1%)	5 (38.4%)	NS [b]	NS [b]	NS [b]
Positive	7 (46.6%)	2 (15.4%)	4 (30.8%)			

Statistical tests were performed with a Kruskal-Wallis test[a] or a Fisher's Exact Test[b]. Values are considered significant if $P<0.05$. Age is expressed as a median (interquartile range). Menopause, chemotherapy, and BRCA mutations were expressed as percentiles.

Benign: Normal fallopian tubes from prophylactic surgeries or benign conditions from women without STIC or cancer.

NoSTIC: Fallopian tubes from women who have ovarian cancer but the examined fallopian tube do not contain transformed cells.

STIC: Fallopian tubes with STIC lesions from women who have ovarian cancer. NS, not significant.

Supplementary Table 3. Multivariable linear regression analysis showing histopathologic characteristics of fallopian tubes with or without STIC lesions in the fimbria region.

Characteristics	Model 1<br>*P* Value	Model 2<br>*P* Value	Model 3<br>*P* Value
**Number of SCE/total number of epithelial cells**			
STIC vs Benign	0.002	0.049	0.045
STIC vs NoSTIC	0.028	0.030	0.021
**Number of CCE/total number of epithelial cells**			
STIC vs Benign	0.038	0.002	<0.001
STIC vs NoSTIC	NS	NS	NS
**Number of secretory cells within SCE**			
STIC vs Benign	<0.0001	<0.0001	<0.0001
STIC vs NoSTIC	<0.0001	0.001	0.001
**Number of ciliated cells within CCE**			
STIC vs Benign	NS	NS	NS
STIC vs NoSTIC	NS	NS	NS

Model 1 was used for comparisons of the characteristics of the Benign, STIC, and NoSTIC groups using the log-transformed values. The multiple linear regression Model 2 included Model 1 plus the potential confounders of menopause, chemotherapy, and BRCA mutations. The multiple linear regression Model 3 included Model 2 plus age as a potential confounder. Values were considered significant if $P<0.05$. SCE, secretory cell expansion; CCE, ciliated cell expansion; NS, not significant.

Supplementary Table 4. Multivariable linear regression analysis showing histopathologic characteristics of fallopian tubes with or without STIC lesions in the ampulla region.

Characteristics	Model 1<br>***P* Value**	Model 2<br>***P* Value**	Model 3<br>***P* Value**
**Number of SCE/total number of epithelial cells**			
STIC vs Benign	NS	NS	NS
STIC vs NoSTIC	NS	NS	NS
**Number of CCE/total number of epithelial cells**			
STIC vs Benign	NS	NS	NS
STIC vs NoSTIC	NS	NS	NS
**Number of secretory cells within SCE**			
STIC vs Benign	<0.001	<0.001	<0.001
STIC vs NoSTIC	0.003	0.021	0.030
**Number of ciliated cells within CCE**			
STIC vs Benign	0.043	0.028	0.016
STIC vs NoSTIC	NS	0.025	0.035

Model 1 was used for comparisons of the characteristics of the Benign, STIC, and NoSTIC groups using the log-transformed values. The multiple linear regression Model 2 included Model 1 plus the potential confounders of menopause, chemotherapy, and BRCA mutations. The multiple linear regression Model 3 included Model 2 plus age as a potential confounder. Values were considered significant if $P<0.05$. SCE, secretory cell expansion; CCE, ciliated cell expansion; NS, not significant.
