# Supplementary material for: Focal Serous Tubal Intra-Epithelial Carcinoma Lesions Are Associated With Global Changes in the Fallopian Tube Epithelia and Stroma
Source: Front Oncol. 2022 Mar 21;12:853755. doi: 10.3389/fonc.2022.853755 (PMC8977528; doi:10.3389/fonc.2022.853755)
Supplement: Supplementary file 2 [file DataSheet_2.pdf]

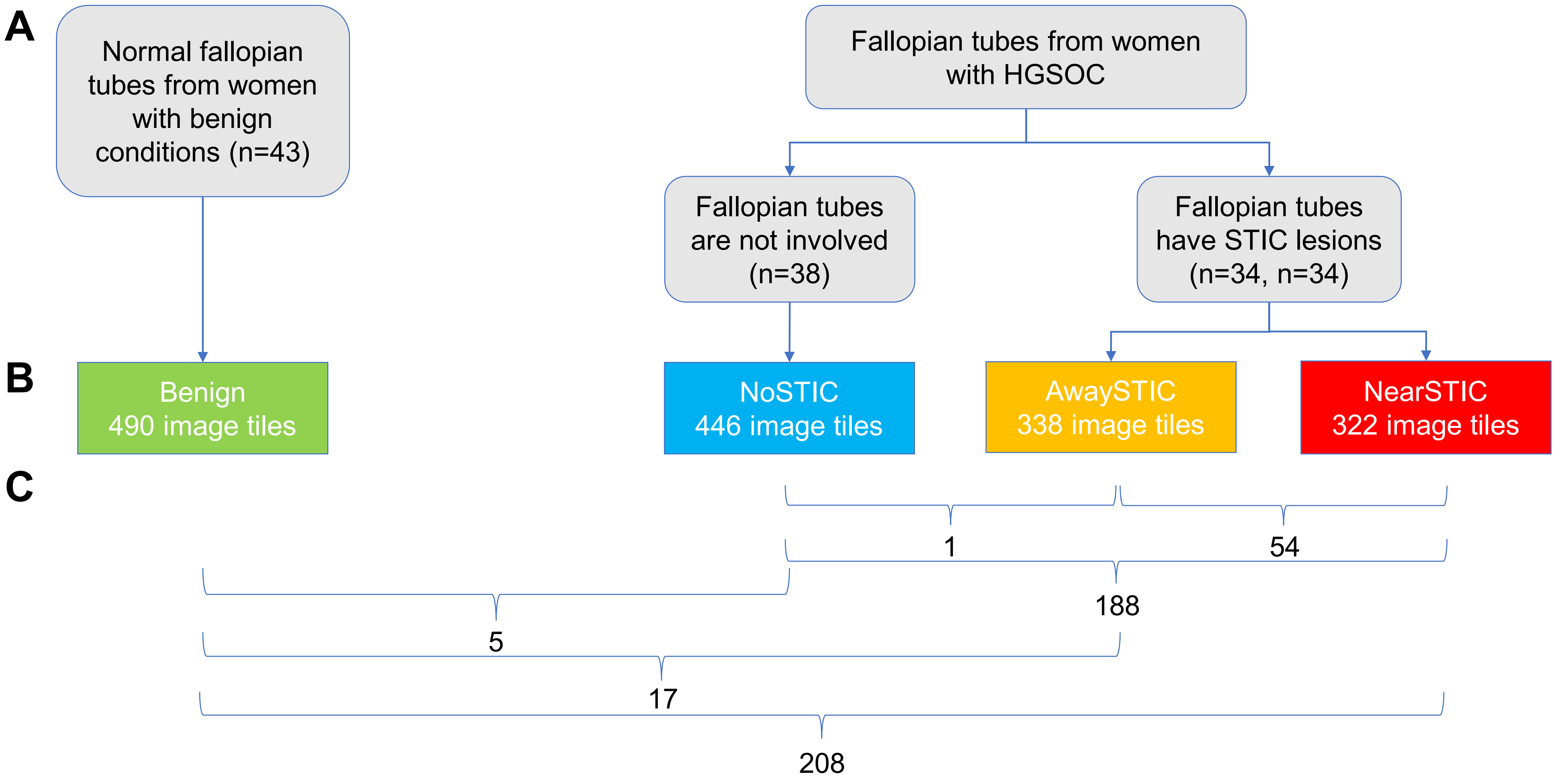

Supplementary Figure 1. A schematic of the breakdown of **(A)** groups of cases, **(B)** image regions, and **(C)** significantly different image features between the groups.

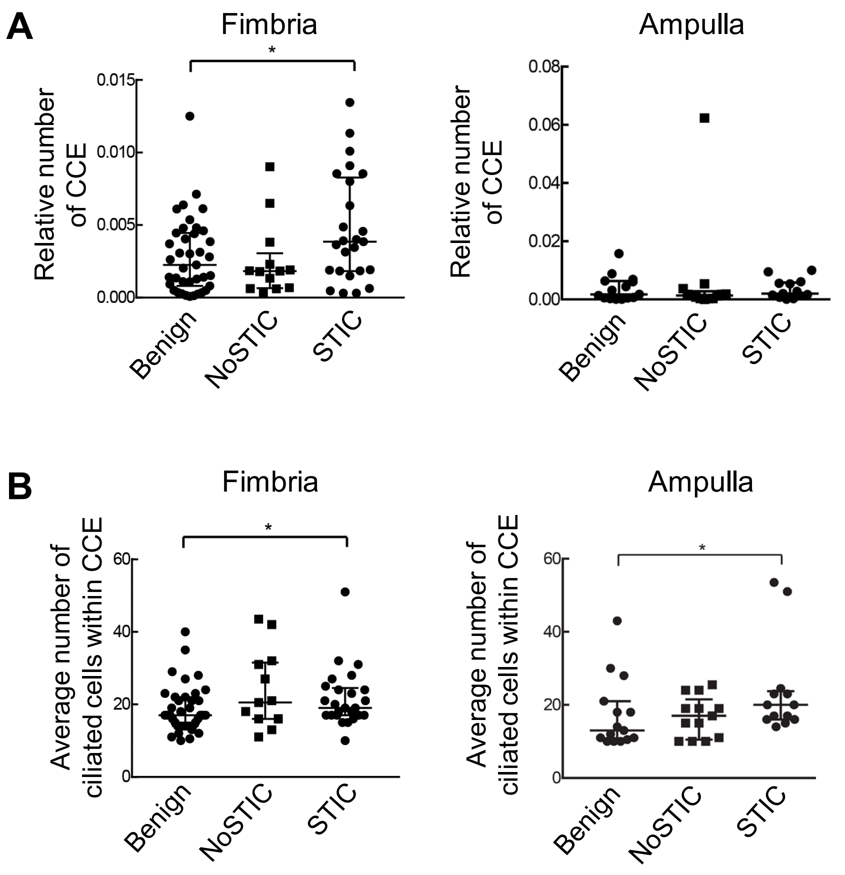

Supplementary Figure 2. (A) The relative number of CCE (number of CCE divided by the total number of epithelial cells in all ROIs on the slide). **(B)** The average number of ciliated cells within CCE. Statistically significant differences were determined using the Kruskal-Wallis test; *$P < 0.05$.

SCE: secretory cell expansion. CCE: ciliated cell expansion. Benign: fallopian tubes from women with benign gynecologic conditions. NoSTIC: fallopian tubes without STIC lesions from women with ovarian cancer. STIC: fallopian tubes with STIC lesions from women with ovarian cancer.

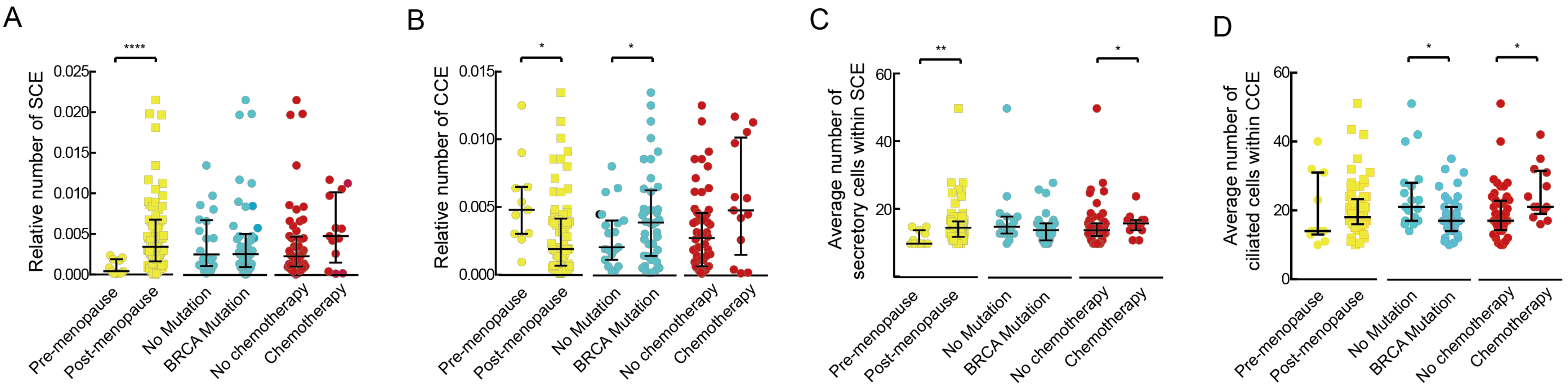

Supplementary Figure 3. The number of **(A)** SCE and **(B)** CCE divided by the total number of epithelial cells in all ROIs on the slide. **(C)** The average number of secretory cells within SCE and **(D)** ciliated cells within CCE. Statistically significant differences were determined using the Kruskal-Wallis test; *$P < 0.05$; **$P < 0.01$.

SCE: secretory cell expansion. CCE: ciliated cell expansion. Benign: fallopian tubes from women with benign gynecologic conditions. NoSTIC: fallopian tubes without STIC lesions from women with ovarian cancer. STIC: fallopian tubes with STIC lesions from women with ovarian cancer.
